# Health technology assessment system in Tanzania: Is it a championing system or still a system lagging behind?

**DOI:** 10.1371/journal.pgph.0004863

**Published:** 2026-03-30

**Authors:** Mwifadhi Mrisho, Fakih S. Bakar, Mohammed Alkhaldi, Patience Vimbayi Mushamiri-Kuzviwanza, Aisha Al Basuoni, Line Enjalbert, Rima Kachach, Maya Hassan, Marcel Tanner, Sara Ahmed

**Affiliations:** 1 Health System Impact Evaluation and Policy, Ifakara Health Institute, Dar es Salaam, Tanzania; 2 Department of Public Health, University of Basel, Basel, Switzerland; 3 The Department of Epidemiology and Public Health, Swiss Tropical and Public Health Institute, Basel, Switzerland; 4 Department of Public Health, School of Health Sciences and Psychology, Canadian University Dubai, Dubai, United Arab Emirates; 5 Faculty of Medicine and Health Sciences, School of Physical and Occupational Therapy, McGill, University, Montreal,; 6 Centre for Outcomes Research and Evaluation (CORE), McGill University Health Center, Montreal, Canada; 7 Centre for Interdisciplinary Research in Rehabilitation of Greater, Montreal (CRIR), The Integrated University Health and Social Services Centre of West-Central Montreal, (CIUSSS West-Central Montreal), Center for Outcomes Research and Evaluation, Clinical Epidemiology, Montreal, Canada; 8 Centre for Tropical Medicine and Global Health, Nuffield Department of Medicine, University of Oxford, Oxford, United Kingdom; 9 School of Public Health, University of the Witwatersrand, Johannesburg, South Africa; 10 Projects Unit, Gaza Community Mental Health Programme, Gaza, Palestine; 11 Faculty of Health Sciences, American University of Beirut, Beirut, Lebanon; University of Milano–Bicocca: Universita degli Studi di Milano-Bicocca, ITALY

## Abstract

Health Technology Assessment (HTA) is a cost-effective solution that decreases inefficiencies and improper investments in health systems. Tanzania has made remarkable efforts in the field of HTA with the establishment of the national HTA committee in 2017; yet, literature on its institutionalization is limited. Using a systems thinking and analytical approach, this study examined Tanzania’s HTA system, focusing on conceptualization, management, implementation, capacity, and use in policy and decision-making. The study was conducted between 2021 and 2023, purposely targeting experts and organizations involved in HTA. Six national HTA experts participated in virtual In-Depth Interviews (IDIs) of HTA from a policy perspective, and eight members of HTA-associated organizations from governmental and non-governmental sectors. Each organization completed one electronic institutional survey to understand HTA from a technical perspective. Findings from both data sources were consistent; half (n = 4) of the participating organisations surveyed affirmed an understanding of the HTA, but the majority showed limited knowledge of a central HTA agency or formal process. The Ministry of Health (MOH) is the main recipient of the HTA reports. Sustainable funding allocated for HTA is limited, and it is irregularly funded by private companies. Key values included safety, cost, economic evaluation, feasibility considerations, and community acceptability. HTA reports were used to inform clinical guidance, healthcare coverage, and pricing decisions. Experts identified the need to strengthen HTA awareness, advocacy, implementation, and institutionalization, supported by political commitment and inclusive governance. Strengthening the Tanzanian national HTA system through a more inclusive national body is essential to address these challenges.

## Introduction

Health Technology Assessment (HTA) is defined as a multidisciplinary and systematic evaluation of properties, effects, and/or impacts of health technologies and interventions. HTA emerged as a response to the urgent need for cost-effective and systematic solutions to address inefficiencies and improper investments in health systems worldwide, particularly in low-resource countries. It evaluates not only medications and public health programmes, but also medical devices, diagnostics, procedures, and any technology or intervention used to promote health, prevent, diagnose, or treat disease [1]. This mechanism is used to generate and disseminate evidence to inform and support policymaking, which makes it a bridge between decision-making and research [2,3]. Although HTA is not a new approach, global interest has increased significantly due to the World Health Organization’s (WHO) emphasis on its role in achieving Universal Health Coverage (UHC) [4] and the Sustainable Development Goals (SDGs), by promoting resource efficiency and equitable health system sustainability [5].

HTA implementation in Low- and Middle-Income Countries (LMICs) that are most in need of stronger guidance for resource allocation remains variable. In Sub-Saharan Africa, HTA capacity ranges from formal institutionalization in some countries to ad hoc or project-based assessments in others, mainly concentrated on pharmacological interventions [3,6]. The lack of institutionalized HTA in regions like Tanzania is attributed to limited operational and human resource capacities. These include inadequate financing for HTA operations, a shortage of specialized technical skills for conducting economic evaluations, and insufficient political will [7]. Tanzania is a unique context due to its active efforts to institutionalize HTA within its health system, aligned with UHC goals. Its practical applications, political commitment, and capacity-building efforts offer valuable lessons for other LMICs.

Tanzania has a gross domestic product (GDP) estimated at around 1,099.3 USD per capita in 2021 [8], and a per capita health expenditure of about US$36.8 [9]. The provision of healthcare is decentralized through the devolution of functions to district and regional governments [10]. At the national level, the Ministry of Health (MoH) plays a key role in the regulation and stewardship of the health sector [10]. The government of Tanzania has been committed to achieving UHC and has introduced many reforms to improve efficiency and equity in healthcare service delivery [3]. Given this political drive, in 2015, the government introduced workshops on the HTA process and applications in low-resource contexts [10].

Tanzania is advancing HTA through a national steering committee, integration into the National Health Insurance Fund (NHIF) benefit design, and alignment with UHC goals. Efforts include capacity building, regulatory use, and local pilot studies to ensure evidence-based and cost-effective health decisions. As part of the process of institutionalizing HTA in the Tanzanian health system, the Pharmaceutical Services Unit (PSU) under the MOH developed the fifth edition of the Standard Treatment Guidelines (STG), and the National Essential Medicines List (NEMLITs) [10]. This development included capacity-building workshops to increase stakeholders’ knowledge of the principles of HTA and evidence-based medicine, which paved the way for convening the first HTA committee in 2017. However, the committee consists mainly of clinicians with limited expertise in health economics and quantitative methods, with only one member having a background in epidemiology and quantitative analysis [11]. There are also considerable unrecognized gaps in HTA understanding, utilization, and practice, compounded by financial challenges, which require proper funding to finance HTA operations in the country. The operational challenges are closely tied to political commitment and financial limitations, including decisions on who will establish the HTA body and its placement within the legislative framework. Overall, there is scarce knowledge and literature on the evolving process of HTA institutionalization in Tanzania. This study seeks to address current gaps by providing a comprehensive analysis of Tanzania’s progress, capacity, and obstacles in HTA institutionalization, and comparing national experience with broader LMIC and global developments.

Consistent with approaches used in similar studies applying systems thinking and analysis [12,13], the overall aim of this study was to comprehensively understand the main pillars of the HTA system in Tanzania and evaluate the current health technologies, services, and processes in the health system through the following specific objectives:

- Assess the understanding level of the HTA concept, importance, and practices among stakeholders;
- Explore the HTA stewardship and governance, capacities, resources, implementation and utilization in the health decision and policy-making process;
- Evaluate the extent to which and how current health technologies, services, and interventions are examined using HTA;
- Identify gaps and propose feasible solutions, such as a framework to support best practices for HTA and knowledge translation strategies in national and regional arenas.

## Materials and methods

Applying an innovative systems thinking approach, this national HTA system analysis was conducted between October 2021 and October 2023 [12,13]. The study used a cross-sectional design employing mixed methods and included an HTA survey informed by a literature review, and a virtual in-depth interview (IDI), as outlined in the protocol [12–14]. The HTA survey was administered to eight organizations involved in HTA to gather information on technical and operational aspects. For the IDI, six high-level experts from various sectors engaged in HTA were interviewed, focusing on policy-related aspects. The following section provides a structured discussion of both data collection tools.

### Study sampling

Two distinct groups of participants were identified based on specific criteria. The first group consisted of eight HTA-associated organizations—governmental, academic, private, or non-governmental—that operate within the health sector in Tanzania. The management of these organizations internally assigned technical/operational team members who run all HTA processes to complete one institutional survey on behalf of their organization. The second group comprised six experts/leaders from these organizations who are responsible for overseeing HTA policies and strategic issues, and who were selected to participate in six individual IDIs.

### Electronic institutional HTA survey

The HTA survey was adapted from the WHO global HTA survey [15]. This electronic survey was used in similar studies [12,13], and it consisted of six domains that cover the pillars of the HTA system, and each domain has relevant items (questions). These domains include understanding of HTA, the use and application of HTA, implementation of HTA, stewardship and management, resources and capacity, and impediments and insights for strengthening HTA. Further domains and questions of this survey on HTA processes, standardization, and HTA and decision-making were developed based on reviewing recent and relevant literature. The survey questions were closed-ended questions to collect. These domains were outlined and recommended by recent and relevant literature and tools developed by leading international organizations specializing in HTA, including the WHO-HTA Unit, HTAi, INAHTA, and EUnetHTA. Each survey administered was completed by the team of the organization involved in HTA. The full survey is enclosed in Supplement 1.

### Virtual in-depth interview

The IDI guide was developed in accordance with best practices for qualitative research [16–18], ensuring a rigorous and systematic approach to data collection. Unlike surveys, which gather standardized responses, the interview questions covered the same HTA domains as the survey, enabling an in-depth exploration of participants’ policy perspectives, strategizing experiences, and decision-making processes. The IDI guide was also used in similar studies [12,13], and is enclosed in Supplement 2. The guide was designed using semi-structured interview techniques, providing a balance between consistency across interviews and flexibility to adapt to emergent themes. Additionally, the guide adheres to recognized reporting standards, such as COREQ (Consolidated Criteria for Reporting Qualitative Research) (enclosed in supplement 3) and SRQR (Standards for Reporting Qualitative Research), enhancing transparency, credibility, and analytical depth in HTA research [19].

Both tools underwent a rigorous review and consultation process involving ten recognized local and international experts across the fields of public health, health systems, digital technology, health economics, epidemiology, clinical specialties, and health policy and management. Feedback from these experts was incorporated into the final versions of the tools.

### Sampling strategy

Sampling and selection process of the organizations and experts was carried out in three phases: mapping and exploration of organizations/experts’ names, initial list preparation, and final eligible list and identification. In the exploration phase, two methods were used to map the names and information of main active organizations and individuals (experts/leaders): 1) a rapid review of grey and published literature, and 2) extensive consultations among research team members and with collaborators in Tanzania. In the preparation phase, an initial comprehensive list that entails twenty names of relevant local and national organizations for the survey and fifteen experts for the IDIs was generated from the two methods. In the identification phase, we set a target to select up to ten existing organizations and up to ten experts. The following inclusion criteria related to organizations: the organization was officially recognized by local and national health authorities, active at least one year since its establishment, had a defined mission for HTA stewardship, production, education, research, and funding; had any previous or current programs, projects, or interventions that directly or indirectly related to HTA; or participated in one or more of HTA activities, were applied. Eight major, active, and relevant HTA-associated organizations from different sectors met these criteria and were selected, while the organizations with no direct or indirect role in HTA and who did not meet the inclusion criteria were excluded. Guided by the application of the predefined selection criteria, a mixed purposive sampling strategies were applied to determine the final list that includes the most relevant organizations. These strategies included critical case, snowballing, convenience, and self-identified sampling by selecting eight critical organizations out of the twenty, where among these eight organizations were accessible, suggested by others, and voluntarily chosen to participate [3,20]. This mixed purposive sampling approach, conducted in consultation with health authorities, led to the selection of eight HTA-associated organizations from the HTA community in Tanzania.

These selected organizations were then contacted, and their representatives were emailed a request to nominate/assign team members involved in technical and operational HTA processes within their organization to complete the survey.

The same sampling and selection approach used for selecting the organizations was also applied to the second group of study participants. Six experts responsible for and working in HTA policy, strategy, and systems were also identified from the same HTA-associated organizations in Tanzania and from international organizations. Those experts were HTA experts, heads of HTA, academics, policymakers, directors, or advisors. The experts with no direct or indirect role in HTA policy, strategy, and systems and who did not meet the inclusion criteria, were excluded.

The research team, led by the Principal Investigator (MA), applied this selection approach of organizations and experts to recruitment to ensure broad agreement and representation across sectors, organizations, and levels of research, policy, managerial, operational, and technical. Given the limited number of HTA communities (organizations and experts working in HTA), the research team set a target sample size of five to ten organizations, and one individual per organization. The sample size was also determined based on availability and accessibility, relevance, diversity, and representation of the essential organizations and experts involved in HTA, taking into account the practical, operational, technical, policy, and scientific considerations raised from the consultations mentioned above.

### Data collection

The electronic HTA survey was designed using McGill’s RedCap^TM^ cloud-based clinical software. Each of the eight HTA-associated organizations completed the survey. This was executed by sending an official invitation outlining the research objectives to each organization’s head for approval. Once approved, the research team distributed the survey via e-mail to the nominated team leader of the organization to guide the technical, operational, practical, and managerial team/staff involved in HTA to complete the survey under the team leaders’ guidance. The research team gave all organizations and the assigned teams a two-month duration to return the completed survey, offering close follow-up, feedback, and support to address any questions or issues related to the survey.

The IDIs were conducted via a web audio-video conferencing platform (Zoom Communications Inc., 2020), and each IDI lasted between 45–60 minutes. Six IDIs were conducted with six experts from policy and strategy levels across different sectors, disciplines, and levels. The PI (MA) and a co-investigator (MM) in Tanzania communicated with all selected experts and coordinated and conducted the virtual IDIs. The PI and co-investigator attended the IDIs, the PI led the IDI and discussion, and the co-investigator facilitated and reported the IDI.

### Data management and analysis

Survey data was analysed using the IBM SPSS Statistics version 29 software program. The survey data were analysed using descriptive statistics, including frequency distribution, percentages, categories, means, and standard deviation. IDI data was audio-recorded and then simultaneously translated and transcribed in English into MS Word sheets by the PI, assisted by trained co-investigators. Transcripts were imported into the software program, MAXQDA 12 (VERBI GmbH, Berlin), for qualitative data management and analysis. Transcripts were checked by the PI to ensure quality. Two coders and co-investigators constructed and validated codes in MAXQDA by classifying transcripts into IDI using a preset coding system that was derived from study objectives. To maintain consistency, a third independent reviewer resolved disagreements. Peer iterative review of themes, participant feedback, and triangulation with survey data were performed by the PI and reviewers to strengthen credibility. The methodological approach of this study was built based on similar studies [16–18]. The Consolidated Criteria for Reporting Qualitative Research (COREQ) approach was followed for reporting the study results [19].

The IDI transcripts were analyzed using thematic analysis, guided by both deductive and grounded theory approaches. The research team used a study framework—developed through expert consultation and literature review—based on six HTA system pillars to ensure systematic and rigorous interpretation.

This study used a triangulated approach to integrate quantitative survey data capturing technical and operational aspects of the HTA system with qualitative insights from expert interviews focused on policy and contextual domains. Data were analyzed separately and then synthesized to identify convergence and divergence. Quantitative trends were contextualized using qualitative findings, enhancing the depth, validity, and credibility of the results and enabling a holistic understanding of HTA system dynamics in Tanzania. To ensure rigor, diverse sampling strategies were used to select participants from various sectors and levels of the health system. Cross-validation across methods confirmed consistent patterns, and steps were taken to minimize bias, as outlined in the recruitment procedures above.

### Ethical consideration

Two ethical approvals were obtained between 2021 and 2023. The first approval was obtained from the McGill Ethics Institutional Board in Canada (Info-Ed File Number: 21-04-009, IRB Number: A04-E13-21A). The second ethical approval was obtained from the Ifakara Health Institute - Institutional Review Board (IHI-IRB) (IHI/ IRB/No: 22–2022), the third was from the Tanzania National Ethical Committee (NatHREC) (NIMR/HQ/R.8a/ Vol.IX/4223). The International Ethical Standards for Biomedical Research Involving Human Persons for implementing this work [21] were followed. Two types of consent were obtained: (1) institutional consent via email from organizational leaders, and (2) individual consent—written (email) or verbal (phone)—for interviews, with a second verbal confirmation given at the start of each interview Participants were informed of their right to withdraw at any time and that their data would be excluded from analysis if they chose to do so.

## Results

### Survey findings on the HTA system from a technical perspective

Eight organizations completed and returned eight surveys. Organizations from the private and NGO sectors were each represented by 38%(n = 3), while academic and other organizations were each represented by 13% (n = 1), including the Tanzanian MoH. With regards to organization classification, 25%(n = 2) of responses represented national and academic/not-for-profit organizations and hospitals, and 13%(n = 1) local organizations.

Survey findings revealed that half of the surveyed organizations (50%) demonstrated a high level of understanding of the HTA purpose and concept, 38% demonstrated a moderate understanding level, and 12% (n = 1) showed a limited understanding. Related to the level of HTA applicability and importance, the bulk of the organizations indicated high (25%, n = 2) to moderate (50%) applicability of HTA in their organizations, and 25% (n = 2) weak and health systems, with a majority (high:12%, (n = 1), moderate: 88%, n = 7). It was reported that the importance of HTA within the respective organizations and health systems was high (87%, n = 7), and moderate (13%, n = 1).

Regarding HTA stewardship and governance, four organizations (50%) confirmed the presence of a central agency responsible for HTA management, while four stated they did not know. Having a formal, well-structured, and appropriately managed process in which HTA information is gathered to support health decision-making, four organizations (50%, n = 4) answered “did not know” and only two organizations (25%) affirmed that they had an HTA formal process, and two (25%) responded that no formal process exists.

Five organizations (62%), who stated that the presence of the HTA process, indicated that they have a legislative requirement considered in the process and results of HTAs in the financing and public health decision-making. The remaining (38%, n = 3) declared a lack of knowledge about the legislation for the HTA process. Three organizations (40%) responded that there was legislation on the role or the status of the HTA process, and its recommendations are mandatory, which must be considered on local and regional evidence, although not bound. While two organizations indicated that this legislation had no formal role in decisions (20%, n = 1), it had partial consideration (20%, n = 1), and the same proportion (20%, n = 1) were not aware of legislation on the role or the status of the HTA process.

**Fig 1:**
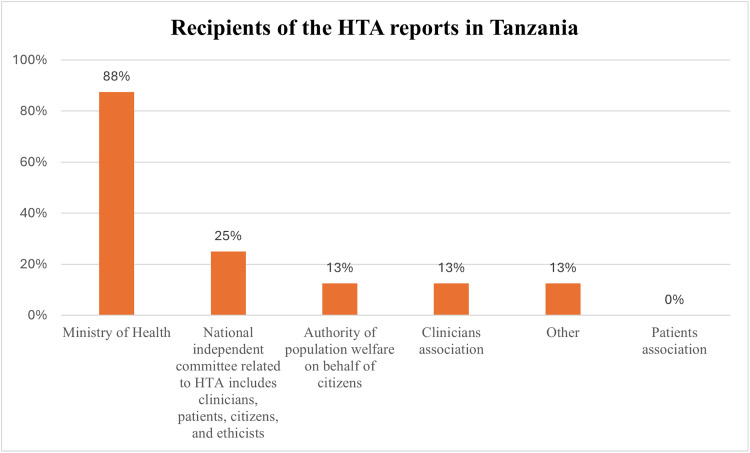
This was a multiple-choice question; % of responses about the recipients of the HTA reports (Responses = 12, Respondents = 8).

Furthermore, survey findings show that only four organizations (50%) knew that there was a national agency/unit/committee that produces HTA reports, mainly a department within MOH, while others didn’t know. Concerning the presence of a unit or committee that produces HTA reports at the subnational level, the majority (88%, n = 7) disclaimed knowledge about this, and only a few (12%, n = 1) affirmed the presence of this committee or body. As illustrated in Fig 1, the majority of the organizations (88%, n = 7) indicated that MoH is the main recipient of HTA reports and other entities such as the national independent committee (25%, n = 2), clinician associations (13%, n = 1), and the authority of population welfare (13%,n = 1), while patients’ associations were not reported as recipients of HTA reports.

**Fig 2:**
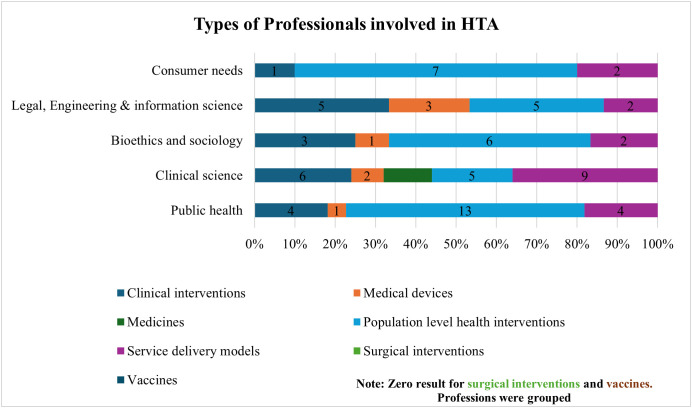
Types of Professionals involved in HTA and its processes.

**Fig 3:**
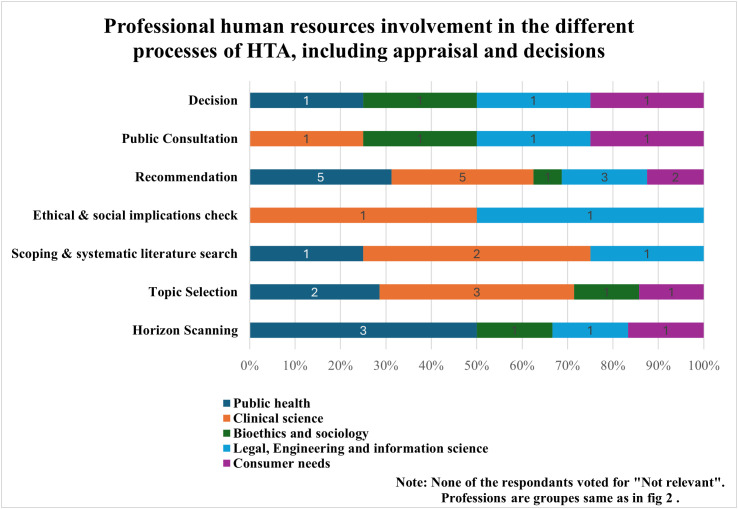
Professionals’ involvement in the relevant stages of the HTA implementation process.

Fig 2 depicts the engagement of various professionals involved in the different processes of HTA, including report preparation, judgment assessment, appraisal, and decisions. Clinical, legal, engineering, and information science professionals were involved in HTA processes, according to participants. Public health, consumer needs, and clinical science were also key inputs in population and service delivery interventions. Fig 3 demonstrates the professionals’ involvement in the HTA implementation process stages that start from horizon scanning to decision making. The recommendation stage was the prominently mentioned relevant stage under public health and clinical science by four and five participants, respectively. Other relevant stages or steps mentioned include horizon scanning and topic selection.

Most organizations (n = 5, 72%) considered external HTA evaluations in their work, while one (14%) reported ‘not available’ and one (14%) ‘not considered’. Regarding conflict-of-interest disclosure, six (67%) agreed on its importance, while three (33%) did not. Only one organization (17%) ensured HTA conclusions were publicly shared or not shared; six (66%) were unaware. Dissemination occurs mainly via online public platforms, organizational websites, and official gazettes, although three organizations were unsure. Regarding policy outcomes, such as efficient resource allocation, social welfare, public confidence, consistent and rational decision-making, improved health outcomes, long-term benefits of innovation, equity, best health practices, and more—one-third of the organizations (33%, n=2) confirmed that these outcomes were based on HTA reports and were made publicly available. The remaining 67% (n=4) disagreed. Only three organizations asserted that civil society provides feedback on recommendations of an HTA report. More than half (57%, n=4) stated their lack of knowledge about the revision process of HTA reports and stakeholders’ involvement.

With regards to resources and capacity needed to support the HTA process, only 12% (n = 1) of organizations indicated that there is sustainable funding, either partial or intermittent funding, or funding itemized in the budget allocated to the HTA. Funding sources included government (with some private contributions), private (with some government support), fully international funding, and contributions from manufacturers.

**Fig 4:**
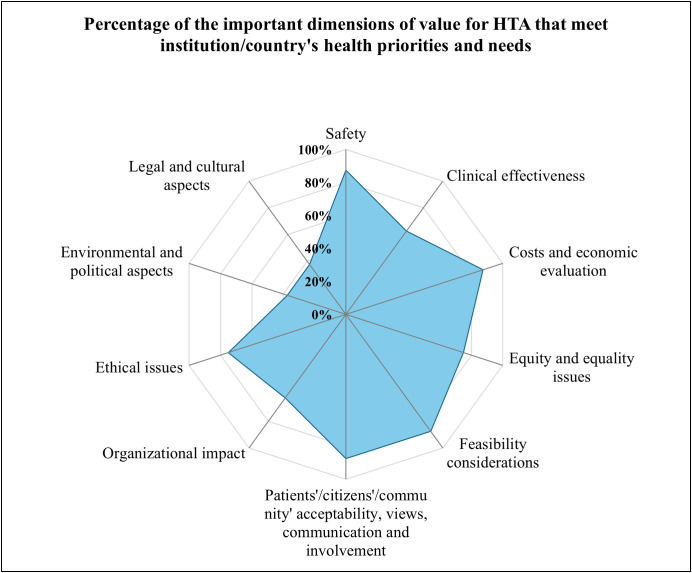
This was a multiple-choice question; Proportion of the most important dimensions of value for HTA that meet the institution/ Country’s health priorities needs (Responses = 10, Respondents = 8).

As illustrated in Fig 4 on key dimensions of value, safety, costs and economic evaluation, feasibility considerations, and patients’/citizen’s/community’ acceptability, views, communication and involvement were the most mentioned (more than three quarters) important dimensions of value for HTA to meet the organization/country’s health priorities and needs. Costs and economic evaluation (70%, n = 7) and clinical effectiveness (65%, n = 6) were moderately considered, while legal and cultural aspects (45%, n = 4) and environmental and political aspects (20%, n = 2) were the least represented. Six Organizations mentioned new technology after the mark authorization was assessed.

**Fig 5:**
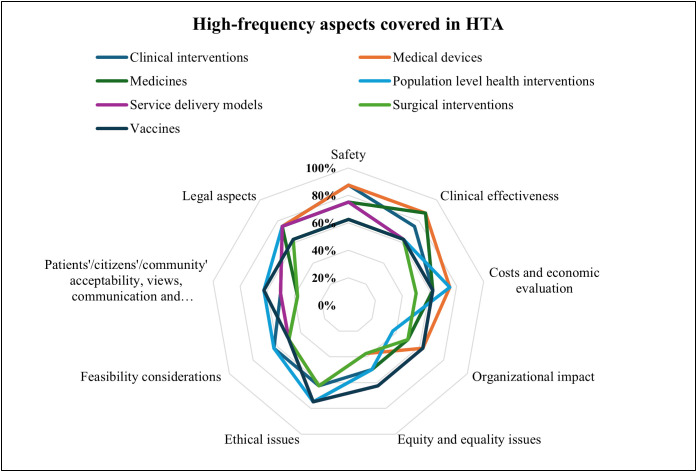
High-frequency aspects covered in HTA in Tanzania (Respondents = 8).

Regarding HTA Coverage (Fig 5), medical devices, clinical interventions, vaccines, service delivery, medicines, and population-level interventions were most frequently assessed, while surgical interventions were moderately covered.

Concerning the availability of guidelines, models, toolkits, and standards (developed by the WHO-HTA Glossary Committee; The International Network of Agencies for HTAINAHTA; EUnetHTA; International Decision Support Initiative (iDSI); the International Society for Pharmacoeconomics and Outcomes Research (ISPOR); Asia (HTAsiaLink), the Americas (RedETSA); and... etc) applied for preparing and developing HTA reports in their organizations, half of the organizations (50%, n = 4) affirmed that there were guidelines and standards applied. Only 38% said that there were no guidelines and standards and 13%(n = 1) were unaware.

**Fig 6:**
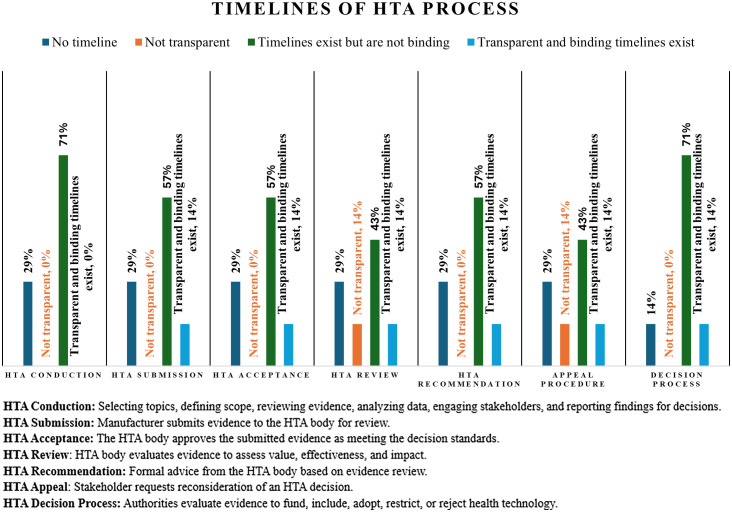
Areas have guidelines and technical practices applied for producing and preparing the HTA report (Respondents = 8 for each area).

Furthermore, Fig 6 demonstrates the areas with guidelines/standards, and technical practices/procedures (recommended by WHO-HTA Glossary Committee; INAHTA; EUnetHTA; iDSI; ISPOR; Asia (HTAsiaLink), the Americas (RedETSA); etc) in place, determined and applied for producing, preparing, and submitting HTA reports in their organizations. Most areas have almost both defined guidelines and practices as follows: clinical interventions (88%), medicines (75%), vaccines (75% Guideline, 62% Technical), medical devices (63% Guideline, 62% Technical), and surgical interventions (63% Guideline, 62% Technical).

**Fig 7:**
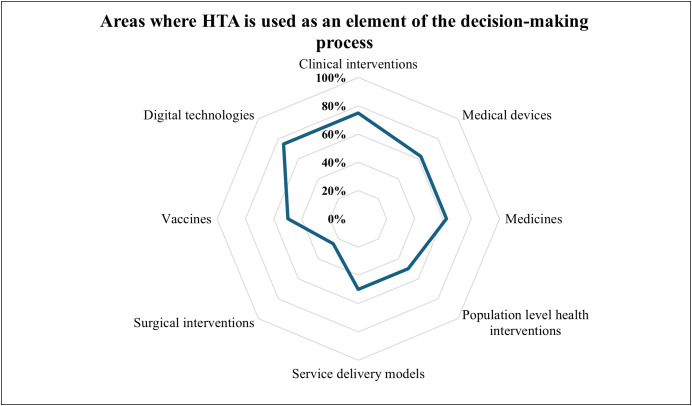
Timelines of the HTA process in Tanzania (Respondents = 7).

Fig 7 presents the timelines for each HTA process step vary, where HTA conduction refers to the implementation of studies following topic selection, HTA submission denotes the formal delivery of HTA reports by manufacturers or stakeholders, HTA review involves expert or committee evaluation of the submitted evidence, and HTA acceptance reflects official endorsement of the findings for policy and decision-making purposes. It shows that timelines exist but are not binding most in HTA conduction (71%, n = 5), submission (57%, n = 4), acceptance (57%, n = 4), review (43%, n = 3), recommendation (57%, n = 4), appeal procedure (43%, n = 3), and decision-making (71%, n = 5). Around 30% of all procedures of the HTA process, plus the decision-making (14%, n = 1), stated there was no timeline. Only 14%(n = 1) declared that the HTA process is transparent, and binding timelines exist, while another 14% (n = 1) declared the process is not transparent.

Regarding the presence of an institutional formal process by which HTA information is gathered to support decision-making on new devices, drugs, vaccines, or interventions, most of the organizations acknowledged that Tanzania has a formal process to support decision-making. Furthermore, support of clinical guidance (75%, n = 6), healthcare coverage (63%, n = 5), and support of pricing decisions (25%, n = 2) were the most frequent purposes of using HTA in the respective organizations.

**Fig 8:**
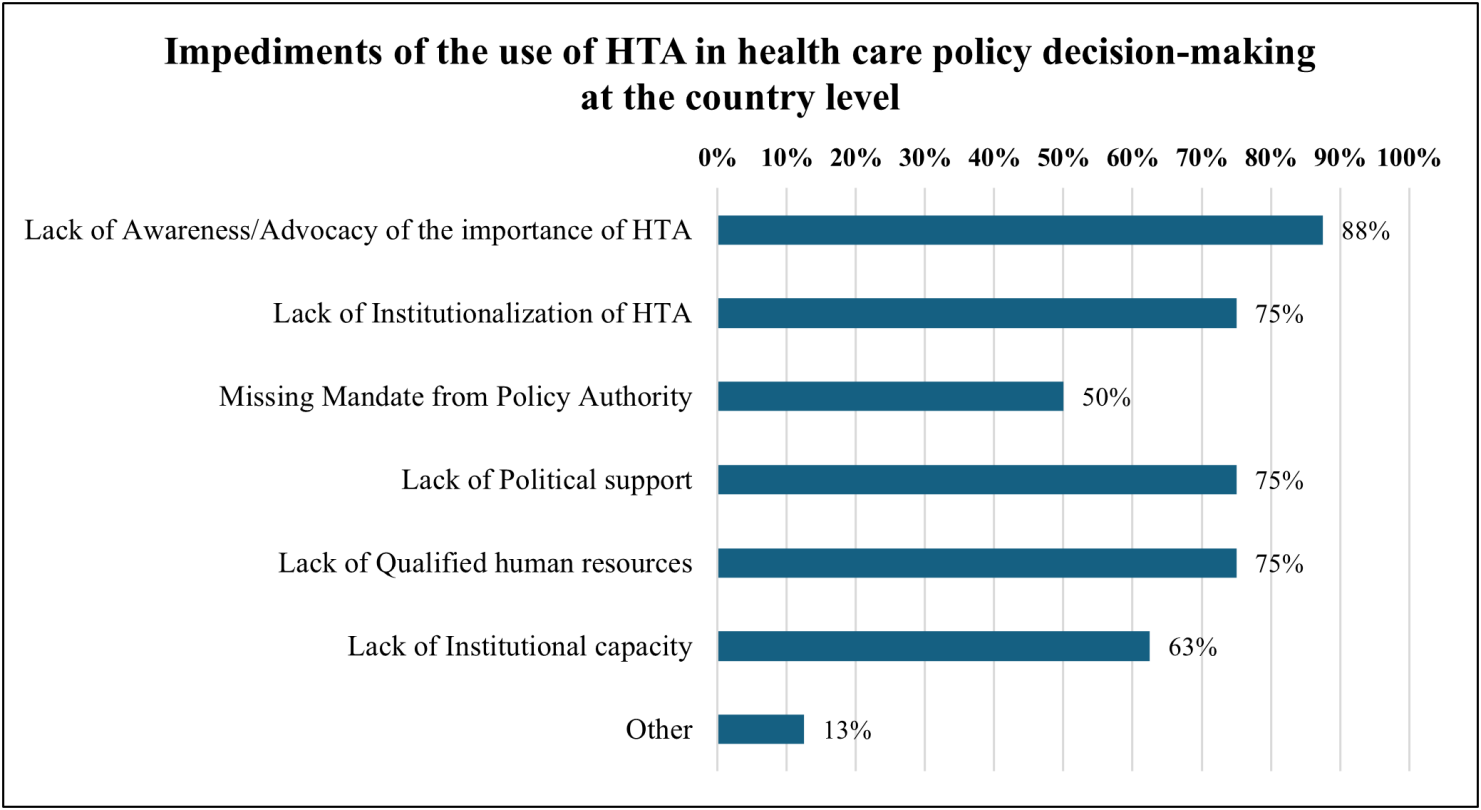
Areas where HTA is used as an element of the decision-making process (Respondents = 8).

With regards to information-gathering practices to support decision-making, seven organizations (88%, n = 7) affirmed that they refer to any information-gathering practices on new interventions (e.g., tests, devices, drugs, vaccines, procedures, programs, or systems) to support decision-making as HTA. The organizations that acknowledged referring to information-gathering practices identified key purposes. Clinical practice guidelines and protocols planning and budgeting (75%, n = 6), pricing of the products (50%, n = 4), benefits and adverse events assessment (50%, n = 4), and indicators of quality of care (50%) were the most stated purposes, while certificate of need and reimbursement (13% each) were the least mentioned. Many organizations (63%, n = 5) responded that the data is collected by the authority, 12% (n = 1) said that it is submitted by the manufacturer, while 25% (n = 2) stated the other. The organizations were also asked to determine areas where HTA is used as an element of the decision-making process, as illustrated in Fig 8. Digital technologies and clinical interventions (75% each), medical devices and medicines (63% each, n = 5), and population-level public health interventions, service delivery models, and vaccines (50% each, n = 4) were the most common areas, while surgical interventions were the least common.

The majority (63%, n = 5) indicated that HTA scientific evidence reports are used on an institutional and national basis. Other organizations stated that (25%, n = 2) of HTA findings are used on a national basis by legislation, while 13% (n = 1) stated that HTA findings and reports are used on a sectoral basis. Lastly, 50% (n = 4) of the organizations indicated that they partially relied on HTA and its conclusions, only 12% (n = 1) completely relied, and 38%(n = 3) confirmed that HTA is the only element of informing the decision.

**Fig 9:**
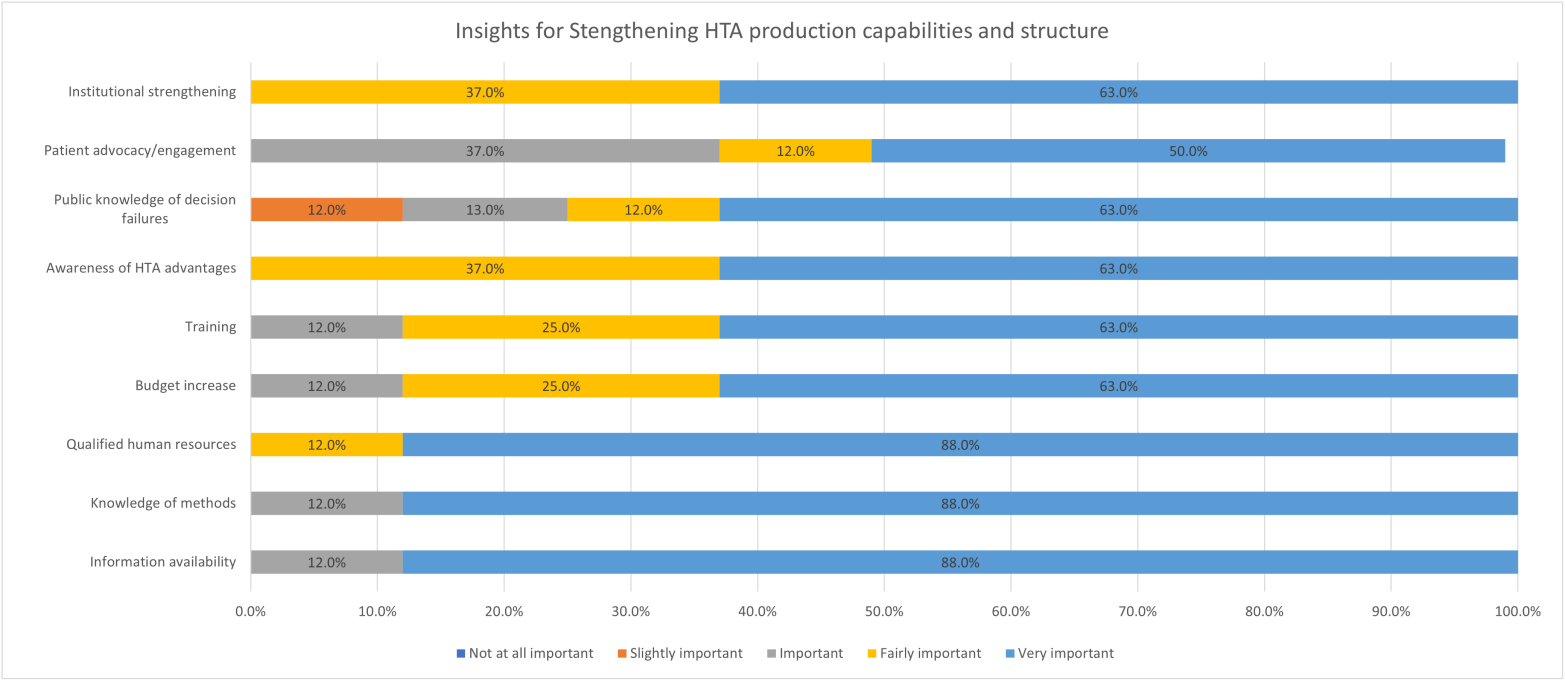
This was a multiple-choice question; impediments of the use of HTA in the healthcare policy decision-making at the country level (Respondents = 8).

Fig 9 presents these impediments which are lack of awareness/advocacy of the importance of HTA (88%, n = 7), lack of institutionalization of HTA (75%, n = 6), lack of political support (75%,n = 6), lack of qualified human resources (75%, n = 6), lack of institutional capacity includes setting up process and well-functioning operations, IT systems supporting HRA, etc (63%, n = 7), and missing mandate from policy authority (50%, n = 4).

**Fig 10:**
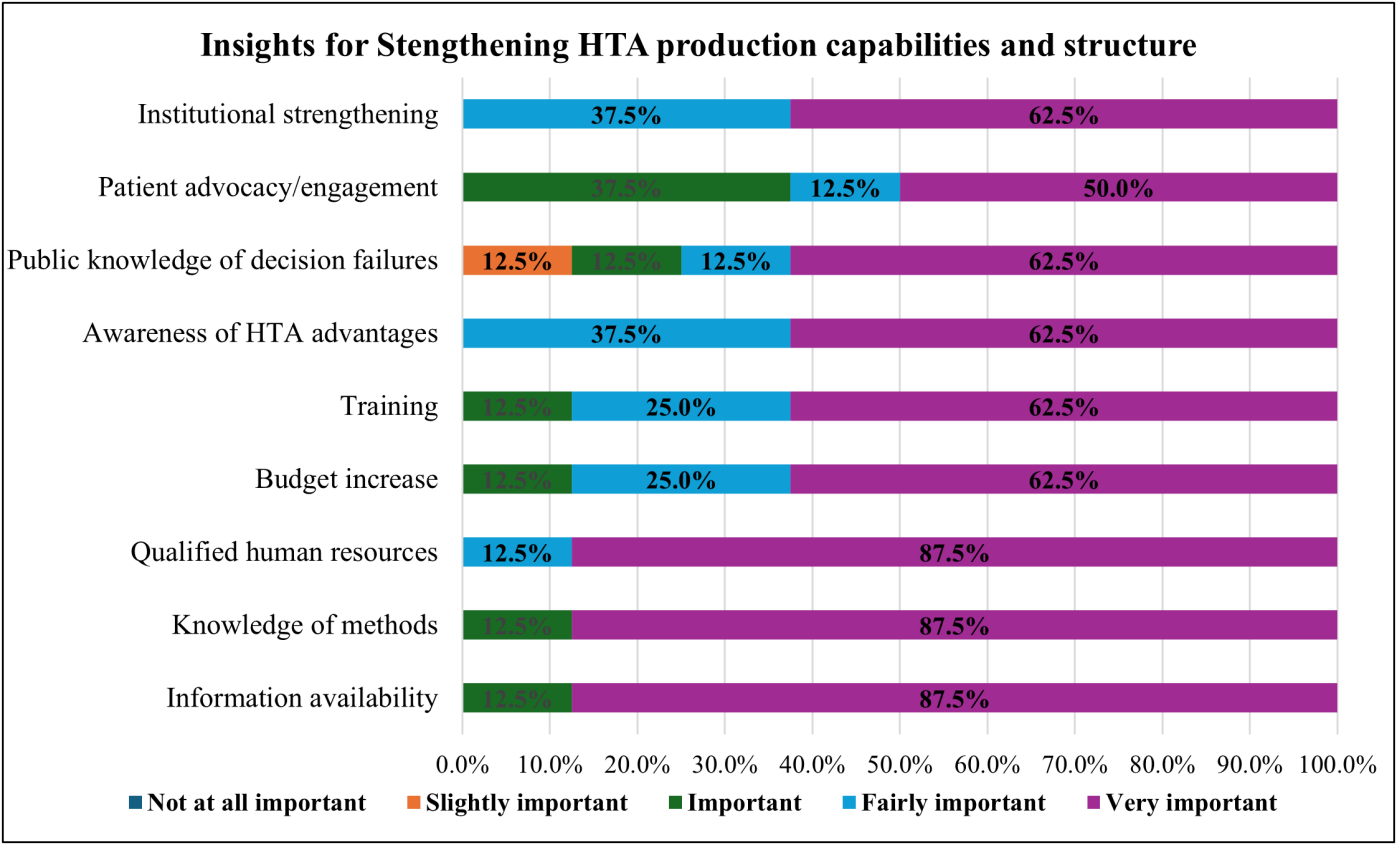
Insights for strengthening HTA production capabilities and structure (Respondents = 8).

For HTA strengthening, the organizations were also asked to provide recommendations and insights on strengthening HTA, as presented in Fig 10. The following were recommended as very important insights, information availability (88%, n = 7), knowledge of methods (88%, n = 7), qualified human resource (88%), budget increase (63%, n = 5), training (63%, n = 5), awareness of HTA advantages (63%, n = 5), public knowledge of decision failures (63%, n = 5), institutional strengthening (63%, n = 5) and patient advocacy/engagement (50%, n = 4).

In terms of enhancing HTA capacity building through academic and training programs, two organizations reported the absence of such programs. Among those that identified existing initiatives, HTA capacity-building efforts were reported through courses, seminars, and workshops (63%, n = 5), internal staff training sessions (25%, n = 2), and higher education programs (13%, n = 1).

### Qualitative evidence on the HTA system: policy perspectives

This system analysis study examined the policy perspectives on HTA of six interviewed experts, including three academics, two MoH seniors, and one private expert.

#### Common understanding and perceived importance of HTA.

Experts uniformly described HTA as a systematic, evidence-based process for analyzing and introducing new technologies before adoption, with the goal of guiding policy and resource allocation. While most agreed on HTA’s importance for healthcare innovation, several highlighted a gap between the perceived potential of HTA and its limited practical application in Tanzania. Concerns were raised about possible unintended consequences, such as ineffectiveness and inequity, if there is no careful implementation and monitoring.

#### Anticipated benefits and impacts.

Experts frequently cited informed decision-making and policy improvement as anticipated benefits of HTA. They believed that HTA could lead to more evidence-based policies and effective healthcare policies, optimizing resource allocation and improving patient outcomes. Other anticipated benefits included improved health outcomes, equitable healthcare access, and greater opportunities for introducing new innovations.

#### Governance, policy frameworks, and political will.

Most experts emphasized the need for strong government support in providing a conducive environment for the development of HTA. Political and policy frameworks were perceived as critical in creating a conducive environment in which relevant stakeholders can cultivate collaborative relationships, facilitate open communication, and collectively contribute to the decision-making process, implementation, and acceptance of new health technologies. While all interviewed experts acknowledged the existence of an HTA governance body in Tanzania, a majority of them were unable to specify its exact title or affiliation, illustrating a lack of clarity and awareness. Two mentioned the MoH as a key actor in governing HTA, emphasizing its role in ensuring medical safety. As one public sector expert stated: “In fact, I cannot say yes. But I’m sure there is an organization which is responsible for HTA” (T6).

#### Capacity constraints and resource challenges.

Experts identified significant constraints in environmental, organizational, and individual capacity for HTA, largely due to fragmented regulations, limited funding, and insufficient infrastructure. An expert stated, “We have a few. Human resources are available in the market but are not absorbed and tapped due to financial resources” (T1). Challenges included a lack of awareness among users, fragmented laws, and limited opportunities for training and skill utilization. The need for enhanced governance, user education, and a skilled workforce, as well as better intersectoral collaboration and sustainable funding, were greatly stressed. Additionally, the importance of adapting HTA to local contexts and leveraging both traditional and digital communication channels was also noted.

#### Education, training, and human resources.

A lack of training opportunities and collaboration with academic institutions was seen as a major barrier to developing a robust HTA workforce. While intellectual resources exist, their supply is limited, and there are restricted opportunities and low demand for HTA-trained professionals. Intersectoral collaboration in HTA education and professional training was viewed as critical for advancing HTA capacities.

#### HTA performance, processes, and evidence use.

Regarding HTA performance, processes, and evidence use, findings revealed that experts generally understood the HTA evaluation process and could describe products or interventions undergoing assessment. The process was viewed as positive, with strengths such as clear guidelines and direction for adapting technologies. However, weaknesses included slow policy translation, the need for political buy-in, and gaps in management and technical capacity. One MoH official highlighted, *“The decision-making is not just a political will granted. It’s 50/ 50. I mean, if political levels are not informed, and if maybe they don’t understand, the technical people don’t understand if they were not granted” (T4).* Satisfaction with HTA evidence sharing varied: Three experts complimented accurate conduct and effective ethics commissions, while others (three experts) pointed to a lack of created research and inconsistent dissemination. *“Yes, so far, the dissemination and use of HTA is so good. The only thing I would say that, in* *my opinion, is the research that is being conducted they are not really innovative research. The implementation research is important and so far, the way this is conducted is proper; it’s super because we have very effective ethical commissions, and we do review both” (T1)*.

#### Communication, advocacy, and evidence use.

Most experts (5 out of 6 experts) agree that HTA evidence is considered in both organizational and national decision-making, especially when adopting new interventions. *“HTA for decision making is linked to the generation of evidence, you know, decision making is an evidence exercise. You need decisions that are made depending on whether this is a need or not, and how you know if this is a need or not. You need to look at the data”* (T5). Experts called for improved mechanisms to ensure HTA information reaches all relevant stakeholders, including through government structures and multiple communication channels.

#### Capacity building and sustainability.

Capacity building emerged as a universal concern, with experts highlighting the need for both short- and long-term initiatives to improve skills and knowledge among HTA practitioners. Financial and technical support, especially from external donors, was seen as crucial for sustaining HTA activities. It is imperative that adequate and continuous funding is ensured for the long-term sustainability for HTA in Tanzania.

## Discussion

This study provides the first comprehensive national analysis of the HTA system in Tanzania, applying a systems thinking and analytical approach. Its relevance lies in its alignment with ongoing reforms in national health insurance, health financing, and universal health coverage (UHC). This study has documented substantial progress in HTA processes since 2017–2018, including the establishment of a formal HTA committee and integration into the NEMLIT revision process [10,22]. However, despite these advances, our findings reveal persistent gaps that hinder full institutionalization and functionality of HTA, underscoring challenges common in LMICs yet with contextual nuances that are unique to the Tanzanian context. It offers critical insights into the current status, achievements, gaps, and future directions of HTA institutionalization in the country.

A comprehensive understanding of the HTA system in Tanzania was achieved through the triangulation of findings from both the survey and expert interviews. The survey captured technical and practical aspects from the perspectives of participating organizations, while the interviews provided deeper insights into policy and contextual dimensions. This integration ensured that expert perspectives were interpreted alongside quantitative trends. Such triangulation strengthens the validity, depth, and credibility of the analysis by combining numerical data with contextual understanding, enabling a more nuanced interpretation of HTA domains within complex health system settings. There are still substantial organizational and national efforts required to sustain and advance these developments to address major challenges. 88% of respondents cited lack of awareness, suggesting the need for national advocacy campaigns, inclusion of HTA in training curricula for policymakers, and targeted communication strategies at the institutional level. This was also highlighted in the qualitative data, where experts mentioned the capacity building for the HTA team. This gap between conceptual understanding and operationalization indicates that without standardized procedures and stronger integration into existing decision-making pathways, HTA risks remaining theoretical rather than transformative. These findings suggest that while awareness of HTA’s purpose exists, operational uptake remains uneven, potentially limiting system-wide impact. This is consistent with other studies highlighting HTA’s growing importance in enhancing health surveillance, system management, education, clinical decision-making, and supporting behavioural change aligned with public health priorities and chronic disease management [14,23–25].

Moreover, there was a clear awareness of the potential benefits of applying HTA, including improved decision-making, health outcomes, equitable access and innovation, while noting that in its absence, there is a heightened risk of adopting ineffective technologies and deepening health inequalities. Leveraging these benefits requires greater political support and government commitment to HTA implementation and institutionalization. Tanzania made significant progress towards institutionalization, while South Africa is still journeying towards establishing a Ministerial Advisory Committee on HTA for National Health Insurance (NHI) and an independent HTA agency [26]. Another experience from Thailand, where HTA has been systematically institutionalized over the past two decades, demonstrates that sustained political commitment, clear governance structures, and active engagement of diverse stakeholders are essential enablers for integrating HTA into health policy and resource allocation [27]. These factors have enabled Thailand to establish a robust and transparent HTA system that informs equitable and efficient healthcare decision-making [27]. Building on this example, strengthening political will and governance mechanisms in Tanzania could similarly enhance the uptake and impact of HTA within its own health system.

As highlighted earlier, some organizations and experts were not aware of the presence of a central agency responsible for HTA management in the country, as shown elsewhere [16]. Qualitative evidence presented a positive understanding of the legislation, possibly due to the high level of expert involvement. In reality, a formal HTA committee was formed previously in Tanzania and should be recognized by all stakeholders. The governance, political, and legislative framework is needed to develop and implement a functional and robust national HTA system [10].

WHO’s HTA guide outlines key elements like a mandate, legal framework, institutional setup, assessment procedures, and monitoring [28]. Tanzania partially reflects this through COSTECH, MoH, and TMDA, with THTAC—led by MoH and involving NGOs and academia—advising on technology reimbursement to support UHC by 2030 [11].

The shortage of HTA expertise reflects broader structural issues in Tanzania’s health workforce development. As 65% of respondents reported insufficient institutional capacity, structured capacity-building programs—such as specialized training in health economics, regional exchange platforms, and mentorship initiatives—are needed to address these shortages. Most professionals involved in HTA lack specialized training in health economics and related fields, reflecting a common challenge in low- and middle-income countries (LMICs) where HTA expertise remains limited [15,25,28].

There was strong consensus among stakeholders on the value of HTA in improving health outcomes, decision-making, equity, and innovation. However, HTA’s implementation remains inconsistent across health areas such as devices, vaccines, and pharmaceuticals [21,24] While the process was deemed appropriate and aligned with country priorities, particularly regarding safety, cost-effectiveness, feasibility, and community acceptability, broader engagement and standardization are still required [28].

HTA reports demonstrated positive practices such as conflict-of-interest disclosures and inclusion of key policy dimensions like social welfare and equity. Since nearly half of stakeholders emphasized weak transparency mechanisms, adopting open-access HTA reports, public consultations, and multi-stakeholder dialogues could enhance legitimacy and uptake. Nonetheless, transparency in report dissemination and civil society engagement remain weak [11,29]. Improving transparency and participation would likely enhance HTA legitimacy and uptake, consistent with experiences in other settings.

Resources and capacity gaps remain the most persistent barriers to HTA system effectiveness in Tanzania. Qualitative and quantitative findings converge on this key point. Key constraints include limited funding, shortages of skilled personnel, fragmented legal and governance frameworks, and weak technical infrastructure. Funding remains primarily dependent on government and private sources, with minimal international support, raising sustainability concerns [10,23,25]. Building the ability to produce and utilize HTA outputs is also essential to its sustainability, and this entails evaluating organizational resources, regulatory frameworks, and individual capabilities [30]. Addressing these sustainability concerns related to HTA in Tanzania requires diversified funding through government budgets, donor support, private contributions, and public-private partnerships, with dedicated budget lines for consistent financing. Political buy-in can be strengthened by engaging policymakers early, embedding HTA in legislation, promoting advocacy through champions, and regularly communicating HTA’s impact. Building a strong training and education system involves developing formal academic programs, offering continuous professional development, fostering collaborations with international HTA networks, and supporting mentorship opportunities to cultivate skilled professionals. Together, these strategies will support a sustainable, well-funded, and politically supported HTA system. All these solutions aligned with solutions proposed by the organizations, where 62% revealed that an increase in budget, training, and awareness is essential.

Findings revealed that while some organizations apply HTA, its full integration into the policy cycle is rare. With 52% noting limited policy guidance, the development of a unified national HTA framework and adaptation of existing WHO/LMIC models could help standardize processes and strengthen institutionalization. Stakeholders called for more robust mechanisms to ensure effective HTA evidence sharing, review, and use. A unified national framework incorporating legal, ethical, and operational components would help standardize and institutionalize HTA [7,10,15] Institutionalized HTA systems demonstrate that such frameworks improve process consistency and credibility, fostering greater evidence use.

Generally, the survey findings revealed varied levels of understanding and application of HTA, with half showing high awareness and most recognizing HTA’s importance in decision-making. While some organizations confirmed formal HTA processes and legislative support, many were unaware of governance structures or specific HTA units, especially at subnational levels. Professionals from multiple disciplines were involved across HTA stages, with common use in clinical interventions, medical devices, and public health programs. Key challenges included limited funding, lack of qualified personnel, insufficient political support, and weak institutionalization. Qualitative findings highlighted consensus on HTA’s value for evidence-based policy and resource allocation but underscored gaps in practical implementation, governance clarity, capacity constraints, and the need for stronger political will, training, and sustainable funding. Experts stressed improving communication, advocacy, and multi-sector collaboration to enhance HTA’s role in health system decision-making and sustainability in Tanzania.

To strengthen the national HTA system, several strategic priorities must be addressed:

- **Capacity Building**: Design and expand education, training, and mentorship programs in academic and public health organizations on HTA, health economics, value-based healthcare, clinical research, epidemiology, and health policy and systems, drawing on successful models from other LMICs. There should be regular professional development workshops to build skilled personnel, and more involvement of external scientists and experts to develop capacities within the current HTA committee.
- **Governance**: Establish a transparent, accountable HTA governance framework with defined procedures, timelines, clear stakeholder roles, and timelines to reduce existing inefficiencies. This framework should entail legal, legislative, administrative, and technical aspects to ensure a functional HTA system. [10].
- **Awareness and Engagement**: Launch targeted advocacy campaigns to increase HTA understanding among policymakers and the public, and actively involve stakeholders, including civil society, in HTA planning, report dissemination, and decision-making processes [6,10,11,22,31].
- **Funding**: Secure dedicated government budget allocations for HTA, complemented by donor and private sector support, ensuring stable and sustainable financing for HTA functions. This could also be achieved through domestic investment, resource mobilization and allocation for a more robust and resilient HTA and health systems [32].
- **Evidence Use**: develop clear institutional mechanisms and guidelines for integrating HTA evidence into policy decisions and improve transparent dissemination of HTA reports to all relevant stakeholders. Connecting HTA outputs to the specific decision-making priorities of the health system and UHC goals offers the strongest pathway to establishing a functional and sustainable HTA system in Tanzania [10].

These priorities are echoed in existing literature on HTA development in LMICs [10,33,34] and align with WHO guidelines for HTA systems.

## Conclusion

This system thinking and analysis approach provides valuable insights into the development of HTA in Tanzania by identifying its key strengths, limitations, and future directions. The main strengths lie in the growing political commitment to evidence-based decision-making, the emergence of institutional capacity, and the increasing awareness and engagement of stakeholders. However, several limitations persist, including limited stakeholder understanding of HTA processes, fragmented implementation, weak institutional structures, and inadequate financial and political support. To address these challenges, the study recommends concrete actions at the policy, practice, and research levels. Policy efforts should focus on embedding HTA within national health legislation, ensuring sustainable government funding, and establishing inclusive mechanisms for multi-stakeholder participation. In practice, institutionalizing HTA units, investing in capacity-building programs, and integrating HTA outputs into health planning and budgeting cycles are essential next steps. From a research perspective, continuous monitoring of HTA system maturity, evaluating the impact of capacity-building models, and identifying strategies to enhance political and public buy-in are crucial. Achieving these recommendations will require sustained commitment, coordinated collaboration, and long-term investment to strengthen HTA systems in Tanzania and similar low- and middle-income settings.

## Supporting information

S1 File Survey Form. (PDF)

S2 File Interview Guide. (DOCX)

S3 File COREQ table. (DOCX)
