# Supplementary material for: Health technology assessment system in Tanzania: Is it a championing system or still a system lagging behind?
Source: PLOS Glob Public Health. 2026 Mar 30;6(3):e0004863. doi: 10.1371/journal.pgph.0004863 (PMC13035126; doi:10.1371/journal.pgph.0004863)
Supplement: S1 File — (PDF) [file pgph.0004863.s001.pdf]

Application of Health Technology Assessment (HTA) In High, Middle and Low-Income Countries

Introduction

I. The overall purpose of the study

School of Physical and Occupational Therapy at McGill University runs and funds this study to develop a comprehensive understanding of HTAs in seven countries ((Canada, Switzerland, Brazil, Lebanon, Jordan, Palestine and Tanzania) for strengthening and development purposes. Data and results generated from this survey will be secured in a well-protected institutional system and used only for research use and for HTA systems development in these countries.

II. Objectives

This survey aims to evaluate the intuitional capacities and processes of HTA in these countries to address the demands for reliable information on health technologies and interventions.

III. Scope

Only national or subnational (region/ province/ state) public health entities are included in this survey. It could be a committee, unit, department, agency, universities or clinical and hospital-based settings, NGOs or private entities.

International entities are not in the scope of this survey.

IV. Definitions and important clarifying notes

HTA is "a multidisciplinary process that uses explicit methods to determine the value of a health technology at different points in its lifecycle. The purpose is to inform decision making in order to promote an equitable, efficient, and high-quality health system".

Note 1: A health technology is an intervention developed to prevent, diagnose, or treat medical conditions; promote health; provide rehabilitation; or organize healthcare delivery. The intervention can be a test, device, medicine, vaccine, procedure, program, or system.

Note 2: The process is formal, systematic, and transparent, and it uses state-of-the-art methods to consider the best available evidence.

Note 3: The dimensions of value for a health technology may be assessed by examining the intended and unintended consequences of using a health technology compared with existing alternatives. These dimensions often include clinical effectiveness; safety, costs, and economic implications; ethical, social, cultural and legal issues; and organizational and environmental aspects, as well as wider implications for the patient, relatives, caregivers, and the population. The overall value may vary depending on the perspective taken, the stakeholders involved, and the decision context.

Note 4: HTA can be applied at different points in the lifecycle of health technology (ie, pre-market, during market approval, post-market, and through to the disinvestment of a health technology).
Source (https://www.valueinhealthjournal.com/article/S1098-3015(20)32060-X/pdf)

V. Instructions

The survey is structured in 6 parts:

Understanding and Perceptions of HTA Application. The use and application of HTA in the health-sector decision-making process Implementation Processes of HTA Governance and Management Resources and capacity supporting HTA processes. Interests and impediments to strengthening the capacity VI. Supplementary information

Please provide any additional information or comments at the end of the survey. Should you require assistance, please contact mohammed.alkhaldi@mail.mcgill.ca The submission deadline of the completed electronic survey is one month after you receive it. There are 62 questions in this survey divided into 6 parts. The approximate period of time that will be dedicated to each institution to complete the survey is a month from the time of emailing the electronic questionnaire. (a team from different departments within the institution needs to be formed to collaborate in completing this electronic survey over different rounds and meetings within a defined period of time)

Name of Organization:	________________
Organization sector:	◯ Public sector (Governmental)<br>◯ Private sector<br>◯ Non-governmental<br>◯ Academic institution<br>◯ Other ______
Organization classification:	☐ national<br>☐ local<br>☐ professional society<br>☐ industry<br>☐ academic/not-for profit<br>☐ hospital<br>☐ private insurer/managed care<br>☐ Other ______
Main field of employment:	☐ Medicine<br>☐ Pharmacy<br>☐ Other health care (e.g., nursing, dietetics)<br>☐ Economics<br>☐ Multidisciplinary<br>☐ Other: ______
Country:	________________

PART 1: Understanding and Perceptions of HTA Application

	Very High	High	Moderate	Weak	Very Weak	Don't Know
1.1 How do you rate the level of your understanding of HTA concept?	○	○	○	○	○	○
1.2 How do you rate the level of your understanding of HTA purpose?	○	○	○	○	○	○
1.3 How do you rate the applicability of HTA in your organization and health system in general?	○	○	○	○	○	○
1.4 How do you rate the HTA application in your sector of working?	○	○	○	○	○	○
1.5 How do you rate the level of HTA importance within your organization and health system in general?	○	○	○	○	○	○
1.6 The application of HTA is important and useful in your area of working?	○	○	○	○	○	○

PART 2: The use and application of HTA in the health-sector decision-making process

Does your country have any formal process by which information is gathered to support decision-making on new devices, drugs, vaccines etc.?

◯ Yes
◯ No
◯ I don't know

The purpose of using HTA in your organization? (check all that apply):

☐ coverage
☐ reimbursement decisions
☐ support of pricing decisions
☐ support of clinical guidance
☐ Other: ______

Which model your institution/country is using for HTA initiation and promotion?

◯ Top-Down Approach (emergence of political interest in promoting an HTA starts at a political level (national or regional) descending to the bottom level.
◯ Bottom-Up Approach (research and academic interest that formed a positive interest in HTA among various actors e.g. producers, health professionals, clinicians, decision makers, patients' associations, etc)
◯ Converging Approach (a combination of the 1st and 2nd approaches, top and bottom level players and mutual efforts between political players and researchers.

Who initiates the HTA?

◯ Manufacture
◯ Private
◯ Authorities
◯ Academia
◯ Other: ______

How is an HTA question or area is identified for assessment?

◯ Decision from authorities
◯ Prioritization exercise
◯ Scientific advice and recommendation
◯ Public consultation
◯ Request from manufacturer or industry about new technologies
◯ Other: please specify ______

Do you refer any information gathering practices on new interventions (e.g., tests, devices, drugs, vaccines, procedures, programs or system) to support decision making as Health Technology Assessment (HTA)?

◯ Yes
◯ No
◯ I don't know

For what purpose is this information gathered? Please choose all that apply:

☐ Certificate of need/carte sanitaire
☐ Clinical practice guidelines and protocols
Planning and budgeting
☐ Pricing of health products
☐ Indicators of quality of care
☐ Reimbursement/ package of benefits
☐ benefits and adverse events assessment
☐ Other (please specify): ______

What are the main data sources your institution/country use in HAT?	◯ Primary such as disease registries, observational studies and electronic health records<br>◯ Secondary sources such as electronic databases and search engines searching of the randomized clinical trials, studies, systematic review, metanalyses, gray literature, Web sites, reports, student theses), and hand searching of selected journals or references of key papers<br>◯ Both sources<br>◯ Other methodologies and sources such as qualitative data (interviews, focus group,.. etc)<br>◯ I don't know
How the HTA data are collected?	◯ Submission by manufacturer<br>◯ Submission by representative<br>◯ Collection by authority<br>◯ Other (please specify) ______
Select the areas where HTA is used as an element of the decision-making process. Please choose all that apply:	☐ Clinical interventions (diagnostic measurements and therapeutic interventions including rehabilitation)<br>☐ Medical devices (in-vitro diagnostics and laboratory equipment, imaging equipment, single use devices, assistive devices).<br>☐ Medicines<br>☐ Population level health interventions (prevention and promotion)<br>☐ Service delivery models<br>☐ Surgical interventions<br>☐ Vaccines<br>☐ Digital technologies (e-health/medicine, telehealth/medicine, or any digital interventions)<br>☐ Other (please specify): ______
How are the HTA findings used?	◯ Reports are used on a national basis by legislation (ie, adopted nationally by legislation)<br>◯ Reports are used on a regional basis by legislation (ie, adopted regionally by legislation)<br>◯ Reports are used on a sectoral basis (ie, voluntarily adopted sectorally)<br>◯ Reports are used on a professional basis (voluntarily adopted professionally)<br>◯ Reports are used on an institutional entity basis (ie, voluntarily adopted by institutions)<br>◯ Reports of scientific evidence are used on institutional and national basis (advisable by scientists and scientific institutions)<br>◯ Other, specify ______
Does the organization making the decision rely on the conclusions of the assessment? If the answer yes, please give examples on decisions that more influenced by HTA than others ie reimbursement of new medicines, pricing, acquisition of devices etc	◯ Yes, completely ______<br>◯ Yes, partially ______<br>◯ HTA is only one element of informing the decision<br>◯ No, they make their own decision<br>◯ Other, please specify: ______

PART 3: Implementation Processes of HTA

From the following listed below, what are the most important dimensions of value for HTA that meet your institution/country's health priorities and needs? Please check all that apply below:

- ☐ Safety
- ☐ Clinical effectiveness
- ☐ Costs and economic evaluation (Cost-effectiveness analysis, budget analysis, utilization, unit cost, indirect costs, real world outcomes)
- ☐ Equity and equality issues
- ☐ Feasibility considerations (e.g. availability of: budget , human resources, infrastructure)
- ☐ Patients'/citizens'/community' acceptability (meeting their needs and evidence for relevant outcomes improvement), views, communication and involvement
- ☐ Organizational impact
- ☐ Ethical issues
- ☐ Environmental and political aspects
- ☐ Legal and cultural aspects (regulations, binding rules, licensing and property rights, ... etc)

At what evolutionary stage of a technology is it likely to be assessed? (for drugs, medical devices, and other technologies, check all that apply)

- ☐ Emerging technology (in development, pre-launch)
- ☐ New technology (after Market authorization)
- ☐ Established or widespread practice (widely or frequently used (monthly and annually) after Market authorization
- ☐ Further development of technology (such as value-added medicines, renovating, repurposing, or new evidence generation)
- ☐ Declining use in practice (testing need for disinvestment)
- ☐ At regular intervals (e.g. every 5 years)
- ☐ Other ______

Do you repeat the assessment in regular intervals including after the marketing of the product?

- ◯ Yes (specify these intervals and/or what is the element triggering a reassessment): ______
- ◯ No
- ◯ N/A

Please estimate which of the following aspects are covered in HTA for clinical interventions and how frequently? Please choose the appropriate response for each item:

	Never, almost never (0%- 19%)	A few times (20%-39)	Sometimes (40%-59%)	Frequently (60%-79%)	Always, almost always (80%-100%)
Safety	○	○	○	○	○
Clinical effectiveness	○	○	○	○	○
Costs and economic evaluation (Cost-effectiveness analysis, budget analysis, utilization, unit cost, indirect costs, outcomes)	○	○	○	○	○
Organizational impact	○	○	○	○	○
Equity and equality issues	○	○	○	○	○
Ethical issues	○	○	○	○	○
Feasibility considerations (e.g. availability of: budget , human resources, infrastructure)	○	○	○	○	○
Patients'/citizens'/community' acceptability (meeting their needs and evidence for relevant outcomes improvement), views, communication and involvement	○	○	○	○	○
Legal aspects (regulations, binding rules, licensing and property rights, ... etc)	○	○	○	○	○

Please estimate which of the following aspects are covered in HTA for medical devices and how frequently? Please choose the appropriate response for each item:

	Never, almost never (0%- 19%)	A few times (20%-39)	Sometimes (40%-59%)	Frequently (60%-79%)	Always, almost always (80%-100%)
Safety	○	○	○	○	○
Clinical effectiveness	○	○	○	○	○
Costs and economic evaluation (Cost-effectiveness analysis, budget analysis, utilization, unit cost, indirect costs, outcomes)	○	○	○	○	○
Organizational impact	○	○	○	○	○
Equity and equality issues	○	○	○	○	○
Ethical issues	○	○	○	○	○
Feasibility considerations (e.g. availability of: budget , human resources, infrastructure)	○	○	○	○	○
Patients'/citizens'/community' acceptability (meeting their needs and evidence for relevant outcomes improvement), views, communication and involvement	○	○	○	○	○
Legal aspects (regulations, binding rules, licensing and property rights, ... etc)	○	○	○	○	○

Please estimate which of the following aspects are covered in HTA for medicines and how frequently? Please choose the appropriate response for each item

	Never, almost never (0%- 19%)	A few times (20%-39)	Sometimes (40%-59%)	Frequently (60%-79%)	Always, almost always (80%-100%)
Safety	○	○	○	○	○
Clinical effectiveness	○	○	○	○	○
Costs and economic evaluation (Cost-effectiveness analysis, budget analysis, utilization, unit cost, indirect costs, outcomes)	○	○	○	○	○
Organizational impact	○	○	○	○	○
Equity and equality issues	○	○	○	○	○
Ethical issues	○	○	○	○	○
Feasibility considerations (e.g. availability of: budget , human resources, infrastructure)	○	○	○	○	○
Patients'/citizens'/community' acceptability (meeting their needs and evidence for relevant outcomes improvement), views, communication and involvement	○	○	○	○	○
Legal aspects (regulations, binding rules, licensing and property rights, ... etc)	○	○	○	○	○

Please estimate which of the following aspects are covered in HTA for population level health interventions (public health interventions) and how frequently? Please choose the appropriate response for each item

	Never, almost never (0%- 19%)	A few times (20%-39)	Sometimes (40%-59%)	Frequently (60%-79%)	Always, almost always (80%-100%)
Safety	○	○	○	○	○
Clinical effectiveness	○	○	○	○	○
Costs and economic evaluation (Cost-effectiveness analysis, budget analysis, utilization, unit cost, indirect costs, outcomes)	○	○	○	○	○
Organizational impact	○	○	○	○	○
Equity and equality issues	○	○	○	○	○
Ethical issues	○	○	○	○	○
Feasibility considerations (e.g. availability of: budget , human resources, infrastructure)	○	○	○	○	○
Patients'/citizens'/community' acceptability (meeting their needs and evidence for relevant outcomes improvement), views, communication and involvement	○	○	○	○	○
Legal aspects (regulations, binding rules, licensing and property rights, ... etc)	○	○	○	○	○

Please estimate which of the following aspects are covered in HTA for service delivery models and how frequently? Please choose the appropriate response for each item

	Never, almost never (0%- 19%)	A few times (20%-39)	Sometimes (40%-59%)	Frequently (60%-79%)	Always, almost always (80%-100%)
Safety	○	○	○	○	○
Clinical effectiveness	○	○	○	○	○
Costs and economic evaluation (Cost-effectiveness analysis, budget analysis, utilization, unit cost, indirect costs, outcomes)	○	○	○	○	○
Organizational impact	○	○	○	○	○
Equity and equality issues	○	○	○	○	○
Ethical issues	○	○	○	○	○
Feasibility considerations (e.g. availability of: budget , human resources, infrastructure)	○	○	○	○	○
Patients'/citizens'/community' acceptability (meeting their needs and evidence for relevant outcomes improvement), views, communication and involvement	○	○	○	○	○
Legal aspects (regulations, binding rules, licensing and property rights, ... etc)	○	○	○	○	○

Please estimate which of the following aspects are covered in the HTA for surgical interventions and how frequently? Please choose the appropriate response for each item

	Never, almost never (0%- 19%)	A few times (20%-39)	Sometimes (40%-59%)	Frequently (60%-79%)	Always, almost always (80%-100%)
Safety	○	○	○	○	○
Clinical effectiveness	○	○	○	○	○
Costs and economic evaluation (Cost-effectiveness analysis, budget analysis, utilization, unit cost, indirect costs, outcomes)	○	○	○	○	○
Organizational impact	○	○	○	○	○
Equity and equality issues	○	○	○	○	○
Ethical issues	○	○	○	○	○
Feasibility considerations (e.g. availability of: budget , human resources, infrastructure)	○	○	○	○	○
Patients'/citizens'/community' acceptability (meeting their needs and evidence for relevant outcomes improvement), views, communication and involvement	○	○	○	○	○
Legal aspects (regulations, binding rules, licensing and property rights, ... etc)	○	○	○	○	○

Please estimate which of the following aspects are covered in the HTA for vaccines and how frequently? Please choose the appropriate response for each item

	Never, almost never (0%- 19%)	A few times (20%-39)	Sometimes (40%-59%)	Frequently (60%-79%)	Always, almost always (80%-100%)
Safety	○	○	○	○	○
Clinical effectiveness	○	○	○	○	○
Costs and economic evaluation (Cost-effectiveness analysis, budget analysis, utilization, unit cost, indirect costs, outcomes)	○	○	○	○	○
Organizational impact	○	○	○	○	○
Equity and equality issues	○	○	○	○	○
Ethical issues	○	○	○	○	○
Feasibility considerations (e.g. availability of: budget , human resources, infrastructure)	○	○	○	○	○
Patients'/citizens'/community' acceptability (meeting their needs and evidence for relevant outcomes improvement), views, communication and involvement	○	○	○	○	○
Legal aspects (regulations, binding rules, licensing and property rights, ... etc)	○	○	○	○	○

Are there any guidelines, models, frame works, manuals, toolkits or standards (developed by WHO-HTA Glossary Committee; INAHTA; EUnetHTA; International decision support initiative (iDSI); the International Society for Pharmacoeconomics and Outcomes Research (ISPOR); Asia (HTAsiaLink), the Americas (RedETSA); and.. etc) applied for preparing and developing HTA reports in your institution?

○ Yes
○ No
○ I don't Know

If Yes, please indicate the followed guidelines:

○ Domestic guidelines created and developed by your own institution
○ WHO guidelines developed by HTA Glossary Committee.
○ INAHTA guidelines.
○ EUnetHTA guidelines
○ iDSI guidelines
○ ISPOR guidelines
○ HTAsiaLink guidelines
○ Americas RedETSA guidelines
○ please specify ______

Which of the following areas listed below have guidelines (among of above ones) and applied for producing and preparing HTA report in your institution? Please choose all that apply:	☐ Clinical interventions (diagnostic measurements and therapeutic interventions including rehabilitation)<br>☐ Medical devices (in-vitro diagnostics and laboratory equipment, imaging equipment, single use devices, assistive devices).<br>☐ Medicines<br>☐ Population level health interventions (prevention and promotion)<br>☐ Service delivery models<br>☐ Surgical interventions<br>☐ Vaccines<br>☐ None
Which of the following areas listed below have defined technical practices or procedures (recommended by WHO-HTA Glossary Committee; INAHTA; EUnetHTA; iDSI; ISPOR; Asia (HTAsiaLink), the Americas (RedETSA); and.. etc)) and applied for submitting HTA reports and mechanisms for communicating them in your institution? Please choose all that apply:	☐ Clinical interventions (diagnostic measurements and therapeutic interventions including rehabilitation)<br>☐ Medical devices (in-vitro diagnostics and laboratory equipment, imaging equipment, single use devices, assistive devices).<br>☐ Medicines<br>☐ Population level health interventions (prevention and promotion)<br>☐ Service delivery models<br>☐ Surgical interventions<br>☐ Vaccines<br>☐ None

Timelines of HTA process (Please answer each row by ticking a single choice "checkmark" to one applicable option among the four)

	No timeline	Not transparent	Timelines exist but are not binding	Transparent and binding timelines exist
HTA conduction	◯	◯	◯	◯
HTA submission	◯	◯	◯	◯
HTA acceptance	◯	◯	◯	◯
HTA review	◯	◯	◯	◯
HTA recommendation	◯	◯	◯	◯
Appeal procedure	◯	◯	◯	◯
Decision process	◯	◯	◯	◯

PART 4: Stewardship and Management

Is the formal process by which HTA information is gathered to support health decision-making is clearly structured and appropriately and systematically managed? * Please select at most one answer

◯ Yes
◯ No
◯ I don't know

NOTE: The process can be within a committee, unit, department or an established HTA organization at a national or subnational level, which provides information for policy decisions for priority-setting, selection, benefit packages, medicines for public procurement, clinical practice guidelines and protocols for public health programmes, etc.

In your country, is there a central agency responsible for HTA management? Please select one answer

◯ Yes, please state their names ______, the role of their authority (legislator, regulator, producer, user, financer, evaluator, etc) ______
◯ No
◯ I don't know

Is there a legislative requirement to consider the process and results of HTAs in the financing and public health decision-making process?

◯ Yes
◯ No
◯ I don't know

Legislation on the role or the status of HTA process and the endorsement of its recommendations in decision-making process (one answer for each row is required by putting one checkmark among the columns)

	No formal role in decisions (unlegislated)	Relatively loos and non-transpare nt	Partial consideration	Must be considered but not binding	Mandatory binding (legislated)	Don't know
HTA process	◯	◯	◯	◯	◯	◯
Local evidence	◯	◯	◯	◯	◯	◯
Regional evidence	◯	◯	◯	◯	◯	◯
International evidence	◯	◯	◯	◯	◯	◯
HTA recommendation and advice	◯	◯	◯	◯	◯	◯

Please add references for legislation as relevant to HTA in your country:

____

Is there a national agency/unit/committee that produces HTA reports? Please choose all that apply:

☐ Yes
☐ No
☐ I don't know

What is the institutional entity where HTA is performed? Please choose all that apply:

☐ HTA organization or agency
☐ National committee within the Ministry of Health
☐ National unit or department within the Ministry of Health
☐ Public Insurance agency or coverage body
☐ Other: ______

Who is the recipients of the HTA reports?	☐ Ministry of Health<br>☐ National independent committee related to HTA includes clinicians, patients, citizens, and ethicists<br>☐ Clinicians association<br>☐ Patients association<br>☐ Authority of population welfare on behalf of citizens<br>☐ Other: ______

Please provide as possible, which of the following professional human resources are involved in the different processes of HTA, including reports preparation, judgment assessment, appraisal, and decisions. Please select the column only when applicable.

	Clinical interventions	Medical devices	Medicines	Population level health interventions	Service delivery models	Surgical interventions	Vaccines	Others
Biomedical and /or clinical engineers	○	○	○	○	○	○	○	○
Biostatisticians / Statistician	○	○	○	○	○	○	○	○
Civil society representative	○	○	○	○	○	○	○	○
Economists/ health economists	○	○	○	○	○	○	○	○
Epidemiologists	○	○	○	○	○	○	○	○
Ethicists	○	○	○	○	○	○	○	○
Lawyers	○	○	○	○	○	○	○	○
Librarians/information specialists	○	○	○	○	○	○	○	○
Medical doctors	○	○	○	○	○	○	○	○
Health professional organizations	○	○	○	○	○	○	○	○
Nurses	○	○	○	○	○	○	○	○
Patients representative	○	○	○	○	○	○	○	○
Pharmacists	○	○	○	○	○	○	○	○
Public Health professionals	○	○	○	○	○	○	○	○
Sociologists	○	○	○	○	○	○	○	○
Others	○	○	○	○	○	○	○	○

Please provide as possible, at what relevant stage or step of HTA process the following professional human resources are involved in the different processes of HTA, including appraisal and decisions. (Please select one answer for each row and one answer among choices in the columns)

	Horizon Scanning	Topic Selection	Scoping & systematic literature search	Evidence collection, synthesis, modeling & Review	Ethical & social implications check	Recommendation	Public Consultation	Decision
Not relevant	◯	◯	◯	◯	◯	◯	◯	◯
Biomedical and /or clinical engineers	◯	◯	◯	◯	◯	◯	◯	◯
Biostatisticians / Statistician	◯	◯	◯	◯	◯	◯	◯	◯
Civil society representative	◯	◯	◯	◯	◯	◯	◯	◯
Economists/ health economists	◯	◯	◯	◯	◯	◯	◯	◯
Epidemiologists	◯	◯	◯	◯	◯	◯	◯	◯
Ethicists	◯	◯	◯	◯	◯	◯	◯	◯
Lawyers	◯	◯	◯	◯	◯	◯	◯	◯
Librarians/information specialists	◯	◯	◯	◯	◯	◯	◯	◯
Medical doctors	◯	◯	◯	◯	◯	◯	◯	◯
Health professional organizations	◯	◯	◯	◯	◯	◯	◯	◯
Nurses	◯	◯	◯	◯	◯	◯	◯	◯
Patients representative	◯	◯	◯	◯	◯	◯	◯	◯
Pharmacists	◯	◯	◯	◯	◯	◯	◯	◯
Public Health professionals	◯	◯	◯	◯	◯	◯	◯	◯
Sociologists	◯	◯	◯	◯	◯	◯	◯	◯
I don't know	◯	◯	◯	◯	◯	◯	◯	◯

Do you also consider HTA evaluations conducted by other organizations or countries?

◯ Yes (specify these organizations/countries): ______
◯ No
◯ N/A

Are conflicts of interest declared for those involved in preparing HTA reports?

◯ Yes
◯ No
◯ I don't know

Are the conclusions of HTA reports publicly available and disseminated?

◯ Yes
◯ No
◯ I don't know

Where these conclusions or reports are published or disseminated?

☐ Online public platform
☐ Institution website
☐ Gazette
☐ I don't know
☐ Other, please specify ______

Do the policy outcomes (efficient resources allocation, social welfare, raising public confidence, consistent and rational decisions, health wellbeing improvement, long term benefits of innovation, equity and fairness, best health practices...etc) based on HTA reports become publicly available?	◯ Yes<br>◯ No<br>◯ I don't know
Can civil society feedback on recommendations of an HTA report?	◯ Yes<br>◯ No<br>◯ I don't know
Are stakeholders, including the community, given a chance to review a draft version of the assessment before the report is finalized?	◯ Yes<br>◯ No<br>◯ I don't know

PART 5: Resources and capacity supporting HTA processes

Is there sustainable funding allocated to the HTA (either partial, intermittent funding, or funding itemized in the budget of your institution)?

◯ Yes
◯ No
◯ I don't know

Who funds the health technology assessment?

☐ Entirely government funded
☐ Mainly government funded with some private contribution
☐ Entirely privately funded
☐ Mainly privately funded with some government contribution
☐ Entirely Internationally funding sources
☐ Partially Internationally funding sources
☐ Mainly government funded with HTA producers' contribution
☐ Manufacturer or supplier
☐ Other: ______

In the last 12 months, approximately, how many health technologies or interventions decisions have been assessed by HTA in the public health sector? Estimated number:

____

Please estimate the percentages from the mentioned number according to the following categories:

Clinical interventions ______
Medical devices ______
Medicines ______
Population level health interventions (prevention and promotion) ______
Service delivery models ______
Surgical interventions ______
Vaccines ______
Other ______

Does you institution measure the impact of HTA on decisions?

◯ Measured
◯ Not measured
◯ I don't know

If so, how many decisions follow the recommendations

____

In the past 12 months, which of the organization listed below has requested an assessment? Please estimate the frequency of requests using the checklist below. Please choose the appropriate response for each item:

	Never, almost never (0%- 19%)	A few times (20%-39)	Sometimes (40%-59%)	Frequently (60%-79%)	Always, almost always (80%-100%)
Healthcare professionals	○	○	○	○	○
Public health care provider (i.e. public hospital)	○	○	○	○	○
Industry	○	○	○	○	○
Ministry of Health	○	○	○	○	○
Other government entities (i.e. finance ministry)	○	○	○	○	○
National Regulatory agency	○	○	○	○	○
Public insurance/ Public Reimbursement Agency /National coverage body	○	○	○	○	○
Procurement agency Other	○	○	○	○	○
Other	○	○	○	○	○

Please estimate the number of professional staff involved in the HTA unit/agency/committee (in fulltime equivalents).

○ 1-5
○ 6-20
○ 21-50
○ 51-100
○ >100

HTA requires multidisciplinary expertise. Please provide as possible, which of the following professional human resources contribute to the preparation of HTA reports. Please select the column only if applicable.

	Clinical interventions	Medical devices	Medicines	Population level health interventions	Service delivery models	Surgical interventions	Vaccines	Others
Biomedical and /or clinical engineers	◯	◯	◯	◯	◯	◯	◯	◯
Biostatisticians / Statistician	◯	◯	◯	◯	◯	◯	◯	◯
Civil society representative	◯	◯	◯	◯	◯	◯	◯	◯
Economists/ health economists	◯	◯	◯	◯	◯	◯	◯	◯
Epidemiologists	◯	◯	◯	◯	◯	◯	◯	◯
Ethicists	◯	◯	◯	◯	◯	◯	◯	◯
Lawyers	◯	◯	◯	◯	◯	◯	◯	◯
Librarians/information specialists	◯	◯	◯	◯	◯	◯	◯	◯
Medical doctors	◯	◯	◯	◯	◯	◯	◯	◯
Health professional organizations	◯	◯	◯	◯	◯	◯	◯	◯
Nurses	◯	◯	◯	◯	◯	◯	◯	◯
Patients representative	◯	◯	◯	◯	◯	◯	◯	◯
Pharmacists	◯	◯	◯	◯	◯	◯	◯	◯
Public Health professionals	◯	◯	◯	◯	◯	◯	◯	◯
Sociologists	◯	◯	◯	◯	◯	◯	◯	◯
Others	◯	◯	◯	◯	◯	◯	◯	◯

Does the Ministry of Health or whoever else is the responsible entity commission independent agencies (NGO's, Academics, etc.) to elaborate, prepare, appraise HTA submissions.

- ◯ Yes
- ◯ No
- ◯ I don't know

Is there one or more agency/unit/committee that produces HTA reports for a subnational level?

- ◯ Yes
- ◯ No
- ◯ I don't know

What is the institutional structure of this HTA body?

- ◯ Department or unit within Ministry of Health
- ◯ Department or unit within another public sector body
- ◯ Standalone public sector institution
- ◯ Other academic or research institution
- ◯ NGO (non-governmental organization)
- ◯ Private sector
- ◯ Other ______
- ◯ I Don't know

Please specify details for every national and subnational agency/unit/committee provided HTA services during the last two years.

	agency/unit/committee	Website	Contact Name	Contact e-mail
National Level				
1	______	______	______	______
2	______	______	______	______
3	______	______	______	______
Subnational Level				
1	______	______	______	______

2 ______ ______ ______ ______
3 ______ ______ ______ ______

PART 6: Interests and impediments to build capacity

Please select from the list below, what impedes at country level the use of HTA in health care policy decision-making. The lack of: (Please choose all that apply)

- ☐ Lack of Awareness/Advocacy of the importance of HTA
- ☐ Lack of Institutionalization of HTA
- ☐ Missing Mandate from Policy Authority
- ☐ Lack of Political support
- ☐ Lack of Qualified human resources
- ☐ Lack of Institutional capacity (setting-up process and well-functioning operations, IT systems supporting HTA, etc.)
- ☐ Other: ______

Please select from list below, what would help to strengthen HTA production capabilities and structure? (Please place one single answer "checkmark" for each row)

	Not at all important	Slightly Important	Important	Fairly Important	Very Important
Information availability	○	○	○	○	○
Knowledge of methods	○	○	○	○	○
Qualified human resources	○	○	○	○	○
Budget increase	○	○	○	○	○
Training	○	○	○	○	○
Awareness of HTA advantages	○	○	○	○	○
Public knowledge of decision failures	○	○	○	○	○
Patient advocacy/engagement	○	○	○	○	○
Institutional strengthening (setting-up process and well-functioning operations, IT systems supporting HTA, etc.)	○	○	○	○	○
Other, specify with rate of importance ...	○	○	○	○	○

Are there any academic or training programs in your country to support HTA capacity building? Please choose all that apply:

- ☐ Courses/seminars/workshops
- ☐ Higher education/Masters
- ☐ Internal staff training sessions or workshops
- ☐ None
- ☐ Other: ______

Is there interest in international HTA training and knowledge platform for continuous education on HTA (Please place one single answer "checkmark" for each row)

	Not at all important	Slightly Important	Important	Fairly Important	Very Important
Offering international training on HTA	○	○	○	○	○
Participating in international courses on specific subjects	○	○	○	○	○
Participating in international continuous education on HTA (certification)	○	○	○	○	○
Participating in expert training on HTA (degree)	○	○	○	○	○
International exchange for Hands-on practice	○	○	○	○	○
Other, specify with rate of importance ...	○	○	○	○	○

Final comments Please add any general comments on the survey: __________________________________

Team Members

Team Leader
Institutional Position ______
Age ______
Gender ______
Educational level ______
Years of experience ______
Level of management ______
Email ______

Team Member 1
Institutional Position ______
Age ______
Gender ______
Educational level ______
Years of experience ______
Level of management ______
Email ______

Team Member 2
Institutional Position ______
Age ______
Gender ______
Educational level ______
Years of experience ______
Level of management ______
Email ______

Team Member 3
Institutional Position ______
Age ______
Gender ______
Educational level ______
Years of experience ______
Level of management ______
Email ______

Team Member 4
Institutional Position ______
Age ______
Gender ______
Educational level ______
Years of experience ______
Level of management ______
Email ______

Team Member 5
Institutional Position ______
Age ______
Gender ______
Educational level ______
Years of experience ______
Level of management ______
Email ______

Team Member 6
Institutional Position ______
Age ______
Gender ______
Educational level ______
Years of experience ______
Level of management ______
Email ______
